# Survival of patients newly diagnosed with colorectal cancer and with a history of previous cancer

**DOI:** 10.1002/cam4.4036

**Published:** 2021-06-30

**Authors:** Sandi L. Pruitt, David E. Gerber, Hong Zhu, Daniel F. Heitjan, Bhumika Maddineni, Danyi Xiong, Amit G. Singal, Anna Tavakkoli, Ethan A. Halm, Caitlin C. Murphy

**Affiliations:** ^1^ Department of Population & Data Sciences University of Texas Southwestern Medical Center Dallas TX USA; ^2^ Harold C. Simmons Comprehensive Cancer Center Dallas TX USA; ^3^ Department of Internal Medicine University of Texas Southwestern Medical Center Dallas TX USA; ^4^ Department of Statistical Science Southern Methodist University Dallas TX USA

**Keywords:** clinical trials, colorectal cancer, rectal cancer, survival

## Abstract

Patients with previous cancer are often excluded from clinical trials despite limited evidence about their prognosis. We examined the effect of previous cancer on overall and colorectal cancer (CRC)‐specific survival of patients newly diagnosed with CRC. This population‐based cohort study from the U.S.A. included patients aged ≥66 years and diagnosed with CRC between 2005 and 2015 in linked Surveillance, Epidemiology, and End Results‐Medicare data. We estimated the stage‐specific effects of a previous cancer on overall survival using Cox regression and on CRC‐specific survival using competing risk regression. We also examined the effect of previous cancer type, timing, and stage on overall survival. Of 112,769 patients, 14.1% were previously diagnosed with another cancer––commonly prostate (32.9%) or breast (19.4%) cancer, with many (47.1%) diagnosed <5 years of CRC. For all CRC stages except IV, in which there was no difference, patients with previous cancer (vs. without) had worse overall survival. However, patients with previous cancer had *improved* CRC‐specific survival. Overall survival for those with stage 0–III CRC varied by previous cancer type, timing, and stage; for example, patients with previous melanoma had overall survival equivalent to those with no previous cancer. Our results indicate that, in general, CRC patients with previous cancer have worse overall survival but superior CRC‐specific survival. Given their equivalent survival to those without previous cancer, patients with previous melanoma and those with stage IV CRC with any type of previous cancer should be eligible to participate in clinical trials.

## Graphical abstract

Patients with previous cancer are often excluded from clinical trials despite limited evidence about their prognosis. We examined the effect of previous cancer on overall and colorectal cancer (CRC)‐specific survival of patients newly diagnosed with CRC. Among other findings, we identified that for all CRC stages except IV, in which there was no difference, patients with previous cancer had worse overall survival compared to those without previous cancer.

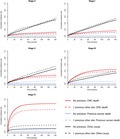

## 1 INTRODUCTION

By 2040, it is estimated that more than 26 million people in the United States (U.S). will be cancer survivors.^1^ Cancer survivors must navigate complex medical needs, including surveillance testing, recurrence, and second cancers. New diagnoses of colorectal cancer (CRC) occur frequently in cancer survivors.^2^ A recent study shows that 15% of older (age ≥65 years) adults diagnosed with CRC, have already survived previous cancer.^3^

As the population of persons with multiple cancers increases,^4^, ^5^, ^6^ clinical trial eligibility criteria continue to exclude cancer survivors newly diagnosed with another cancer.^7^ The majority of National Cancer Institute (NCI)‐sponsored lung cancer (80%) and CRC (76%) trials^8^ have this exclusion.^9^ Recently, the *American Society of Clinical Oncology–Friends of Cancer Research* Clinical Trial Eligibility Working Group recommended against excluding these patients solely on the basis of previous or stable, concurrent malignancy.^10^, ^11^ The Food and Drug Administration also drafted similar guidance.^12^ Yet, despite recent efforts to relax clinical trial eligibility, there are limited data to inform evidence‐based inclusion criteria. Descriptive epidemiologic studies also frequently exclude patients with previous cancer, which makes it even more challenging to draw conclusions about the characteristics and prognosis of this population relative to those without previous cancer.^13^, ^14^ Although some evidence suggests that CRC patients with previous cancer have worse overall survival,^15^ findings are mixed, with some studies documenting improved survival or no difference.^16^, ^17^, ^18^, ^19^ Many questions remain unanswered about the impact of previous cancer and whether the impact varies by CRC stage, cause‐of‐death, or type of previous cancer.

To address these gaps, we used population‐based data from the United States to answer the following research questions among patients newly diagnosed with CRC: Does overall or CRC‐specific survival differ for patients with previous cancer compared to those without previous cancer? and How are characteristics of the previous cancer, including previous cancer type, time elapsed between previous cancer and CRC, and previous cancer stage, associated with overall survival?

Our answers to these important questions should be used to inform design of trial inclusion and exclusion criteria and will help draw attention to the unique needs of the growing population of cancer survivors.

## 2 METHODS

### 2.1 Data

We analyzed U.S. population‐based data from the linked Medicare claims and National Cancer Institute Surveillance, Epidemiology, and End Results (SEER) program.^20^ This study included Medicare patients ≥66 years of age and newly diagnosed with CRC (American Joint Committee on Cancer [AJCC] 6th edition stages 0–IV) between 2005 and 2015. All patients had full coverage of Medicare Parts A and B from 1 year before and 1 year after CRC diagnosis or until death. We excluded HMO members and patients with only autopsy or death certificate records, incomplete dates of diagnosis or death, discrepancies between SEER and Medicare birthdate of 1 year or more, unknown CRC stage, or discrepancies between sequence number and tumor site recode.

### 2.2 Measures

We defined previous cancer using our published approach.^8^, ^21^ We determined order and timing of all primary cancer diagnoses using the SEER variable site recode and associated ICD‐O‐3 values. We restricted our sample to two groups of patients diagnosed with CRC: (a) patients with no previous cancer and (b) patients with one previous cancer of a different type. We excluded patients with more than one previous cancer and/or any previous CRC because cause of death misclassification is more common for patients with more than one previous cancer.^22^ This allowed us to more accurately measure CRC‐specific survival. The excluded patients, when compared to those with one previous cancer of different type, were older, diagnosed at later stage, and more likely to be male.

We examined two outcomes: overall survival and cause‐specific survival. Overall survival was measured as the interval in months between CRC diagnosis and death from all causes. Patients were followed until death date or 31 December 2016. For cause‐specific survival, we defined three possible causes of death: death from previous cancer (possible only for patients with previous cancer), CRC, or any other causes.

We measured numerous covariates. The following patient characteristics were defined at the time of CRC diagnosis: age, sex, race/ethnicity, marital status, region, neighborhood poverty, and urban/rural residence. Patients with Medicaid were identified using the state buy‐in variable.^23^ We measured the following characteristics of the CRC: tumor location, grade, histology, and stage at diagnosis defined using AJCC, 6^th^ edition. Using SEER and Medicare claims, we measured receipt of chemotherapy, radiation, and surgery type. We measured the number of 10 frailty‐defining diagnoses (e.g., falls, dementia, etc.) and 16 non‐cancer comorbidities (e.g., diabetes, cerebrovascular disease, etc.) in the year prior to CRC diagnosis (see Appendix 1).^24^, ^25^, ^26^ For Medicare‐defined covariates, we searched inpatient, outpatient, and carrier claims.

### 2.3 Analysis

We reported prevalence of previous cancer and compared covariates in patients with and without previous cancer using chi‐square tests. Among those with previous cancer, we also described the cancer type, timing, and stage of the previous cancer.

To assess the association of previous cancer and overall survival, we estimated cumulative incidence of death from all causes using unadjusted Nelson–Aalen curves. To account for the effects of potential covariates, we further modeled the hazard rate for overall survival using adjusted Cox regressions including all measured covariates. We also examined the effect of previous cancer type, timing, and stage on overall survival using adjusted Cox regressions. We categorized previous cancer type as follows: no previous cancer or previous breast, urinary bladder or other urinary organs, melanoma, lung, corpus/uterus, lymphoma, oral cavity, prostate, or other. We categorized time elapsed between previous cancer and CRC diagnosis as no previous cancer or <1 year, 1‐5 years, or >5 years. We measured previous cancer stage as no previous cancer or as localized, regional, distant, or unstaged. Hazard ratios (HR) and 95% confidence intervals (CI) quantify the effects of previous cancer on overall survival.

We conducted competing risk analyses as follows. For each cause of death (previous cancer, CRC, and other), we plotted cumulative incidence function curves and estimated adjusted Fine and Gray proportional subdistribution hazard regressions.^27^ We calculated subdistribution hazard ratios (sdHR) and 95% confidence intervals to quantify the effects of previous cancer on CRC‐specific death and death from other causes while accounting for competing risks.

To quantify the absolute differences in prognosis between those with and without previous cancer, we estimated 5‐year risk of death for all causes and separately by cause (CRC, previous cancer, and other causes).

Lastly, we repeated overall survival analyses in a subset of “clinical‐trial type” patients. This subsample included patients with no comorbidity or frailty, who received any CRC treatment, and who were aged <75 years at CRC diagnosis.

Survival analyses were conducted separately by CRC stage. We fitted models with and without adjustment for all covariates. To assess the proportional hazard assumption, we examined log–log plots and Schoenfeld residuals; when violated, we included interaction terms of previous cancer with (log)time. We conducted analyses in SAS 9.4 (SAS Institute Inc.) and created figures in R Studio Version 1.2.5033.^28^ The University of Texas Southwestern Medical Center Institutional Review Board approved this study (STU 042018–032).

## 3 RESULTS

### 3.1 Sample description

Appendix 2 shows sample selection. Of 112,769 eligible patients newly diagnosed with CRC, 15,935 (14.1%) had previous cancer. Table 1 demonstrates that those with and without previous cancer differed in several ways. For example, patients with previous cancer were older, more likely to be male and to receive chemotherapy, radiation, and local tumor excision. Most previous cancers (82.7%) were diagnosed at localized or regional stage. Appendix 3 shows the characteristics of those with and without previous cancer by CRC stage.

**TABLE 1:** Characteristics of patients diagnosed with colorectal cancer between 2005 and 2015 by previous cancer at time of colorectal cancer diagnosis (*N*=112,769)

	No previous cancer (*N* = 96,834)	Previous cancer^a^ (*N* = 15,935)	*p* value
Sex			
Male	44,983 (46.5%)	9151 (57.4%)	<0.001
Female	51,851 (53.5%)	6784 (42.6%)	
Age			
66–70	16,189 (16.7%)	1955 (12.3%)	<0.001
70–75	21,090 (21.8%)	3298 (20.7%)	
75–80	21,115 (21.8%)	3632 (22.8%)	
80–85	19,311 (19.9%)	3689 (23.2%)	
>85	19,129 (19.8%)	3361 (21.1%)	
Race/ethnicity			
Non‐Hispanic White	76,457 (79.0%)	13,140 (82.5%)	<0.001
Hispanic white	3880 (4.0%)	498 (3.1%)	
Black	8872 (9.2%)	1389 (8.7%)	
Other	7417 (7.7%)	879 (5.5%)	
Unknown	208 (0.2%)	29 (0.2%)	
Marital status			
Married/Unmarried or domestic partner	46,154 (47.7%)	8415 (52.8%)	<0.001
Separated/Divorced/Widowed	36,997 (38.2%)	5526 (34.7%)	
Single	8970 (9.3%)	1253 (7.9%)	
Unknown	4713 (4.9%)	741 (4.7%)	
Medicaid			
Yes	18,298 (18.9%)	2120 (13.3%)	<0.001
No	78,536 (81.1%)	13,815 (86.7%)	
Poverty			
0% to <5% poverty	21,936 (22.7%)	4102 (25.7%)	<0.001
5% to <10% poverty	24,961 (25.8%)	4373 (27.4%)	
10% to <20% poverty	28,780 (29.7%)	4393 (27.6%)	
20% to 100% poverty	20,947 (21.6%)	3033 (19.0%)	
Unknown	210 (0.2%)	34 (0.2%)	
Urban–rural indicator			
Metro	79,105 (81.7%)	13,225 (83.0%)	<0.001
Urban	15,440 (15.9%)	2376 (14.9%)	
Rural or Unknown^b^	2289 (2.4%)	334 (2.1%)	
Region			
Northeast	21,237 (21.9%)	3366 (21.1%)	<0.001
South	25,225 (26.0%)	3297 (20.7%)	
Midwest	12,621 (13.0%)	2851 (17.9%)	
West	37,751 (39.0%)	6421 (40.3%)	
Colorectal cancer stage			
Stage 0	6733 (7.0%)	1246 (7.8%)	<0.001
Stage I	22,509 (23.2%)	4204 (26.4%)	
Stage IIA	22,586 (23.3%)	3726 (23.4%)	
Stage IIB	3927 (4.1%)	566 (3.6%)	
Stage IIIA	2454 (2.5%)	383 (2.4%)	
Stage IIIB	13,035 (13.5%)	1938 (12.2%)	
Stage IIIC	7329 (7.6%)	1199 (7.5%)	
Stage III NOS	139 (0.1%)	25 (0.2%)	
Stage IV	18,122 (18.7%)	2648 (16.6%)	
Tumor location			
Proximal colon (cecum, ascending)	36,255 (37.4%)	6208 (39.0%)	<0.001
Transverse colon (hepatic flexure, transverse colon, and splenic flexure)	13,579 (14.0%)	2457 (15.4%)	
Distal colon (descending colon, sigmoid colon, overlapping lesion, and colon NOS)	24,232 (25.0%)	3727 (23.4%)	
Rectum (rectosigmoid junction, rectum)	21,896 (22.6%)	3380 (21.2%)	
Unknown	872 (0.9%)	163 (1.0%)	
Grade			
Well differentiated	7756 (8.0%)	1304 (8.2%)	0.344
Moderately differentiated	56,621 (58.5%)	9196 (57.7%)	
Poorly differentiated	15,510 (16.0%)	2579 (16.2%)	
Undifferentiated	2363 (2.4%)	418 (2.6%)	
Not determined	14,584 (15.1%)	2438 (15.3%)	
Histology			
Mucinous adenocarcinoma	83,836 (86.6%)	13,771 (86.4%)	<0.001
Other adenocarcinoma	8751 (9.0%)	1508 (9.5%)	
Non‐adenocarcinoma	3023 (3.1%)	519 (3.3%)	
Unknown	1224 (1.3%)	137 (0.9%)	
Surgery			
No surgery	13,610 (14.1%)	2251 (14.1%)	<0.001
Local tumor excision	6816 (7.0%)	1347 (8.5%)	
Partial colectomy or proctectomy	31,715 (32.8%)	4926 (30.9%)	
Subtotal colectomy	40,058 (41.4%)	6663 (41.8%)	
Total colectomy or proctectomy	3022 (3.1%)	555 (3.5%)	
Proctocolectomy	785 (0.8%)	86 (0.5%)	
Other/not specified	828 (0.9%)	107 (0.7%)	
Chemotherapy			
Yes	26,871 (27.7%)	5074 (31.8%)	<0.001
No	69,963 (72.3%)	10,861 (68.2%)	
Radiation			
Yes	10,716 (11.1%)	2066 (13.0%)	<0.001
No	86,118 (88.9%)	13,869 (87.0%)	
Comorbidity count			
0	44,464 (45.9%)	6652 (41.7%)	<0.001
1	27,472 (28.4%)	4722 (29.6%)	
2	13,151 (13.6%)	2390 (15.0%)	
≥ 3	11,747 (12.1%)	2171 (13.6%)	
Frailty count			
0	71,383 (73.7%)	11,786 (74.0%)	0.344
1	20,126 (20.8%)	3323 (20.9%)	
2	4283 (4.4%)	654 (4.1%)	
≥ 3	1042 (1.1%)	172 (1.1%)	
Cause of death			
Alive	39,919 (41.2%)	5942 (37.3%)	<0.001
Died of colorectal cancer	33,011 (34.1%)	4072 (25.6%)	
Died of previous cancer	0 (0%)	1185 (7.4%)	
Died of other causes	23,904 (24.7%)	4736 (29.7%)	
Median months (interquartile range) from previous cancer diagnosis to CRC diagnosis	—	65 (25, 124)	—
Stage of previous cancer	—		
In situ/localized/regional	—	12,644 (82.7%)	
Distant	—	1147 (7.5%)	

^a^ Includes patients with one previous non‐colorectal cancer.

^b^ Unknown account for n≤11 per cell and are grouped together to avoid small cell sizes.

Table 2 shows previous cancer type and timing. The most common previous cancers were prostate (32.9%), breast (19.4%), urinary bladder or other urinary organs (6.8%), melanoma (6.4%), and lung cancer (5.7%). The median time between the diagnosis of previous cancer and CRC was 65 months, with roughly half (47.1%) diagnosed within 5 years.

**TABLE 2:** Characteristics of previous cancer, including previous cancer type and time elapsed between diagnosis of previous cancer and colorectal cancer (CRC) among all CRC patients with previous cancer and separately by CRC stage at diagnosis (n = 15,935)

Type of previous cancer	Overall		Diagnosed < 5 years before CRC		Median months between previous and CRC and interquartile range			Colorectal cancer stage				
								0	I	II	III	IV
	*N*	Column %	*N*	Row %	Months	Q1	Q3	Row %	Row %	Row %	Row %	Row %
Anus, anal canal, anorectum	63	0.4	42	66.7	30	2	91	—	25.4	28.6	22.2	—
Bone and joints; soft tissue	76	0.5	38	50	60	21	116.5	—	31.6	27.6	22.4	—
Brain and other nervous system, including cranial nerves and other nervous system	24	0.2	13	54.2	56	22	133	—	—	—	—	—
Breast	3090	19.4	1215	39.3	80	34	145	7.5	25.4	27.0	24.6	15.4
Cervix; vagina; vulva; other female genital organs	173	1.1	66	38.2	94	26	237	6.9	19.1	30.6	21.4	22.0
Small intestine	72	0.5	58	80.6	17	0	51	—	36.1	22.2	20.8	—
Corpus and uterus	720	4.5	231	32.1	102	43	192	4.4	24.7	31.4	23.9	15.6
Esophagus	72	0.5	60	83.3	3.5	0	37	18.1	29.2	23.6	—	—
Eye and orbit	43	0.3	15	34.9	107	39	170	—	—	—	—	—
Kidney and renal pelvis	471	3	265	56.3	51	15	104	10.0	25.7	26.8	22.7	14.9
Liver and intrahepatic bile duct; gallbladder and other biliary; other digestive organs	86	0.5	60	69.8	29	2	72	—	29.1	19.8	17.4	22.1
Lung and bronchus; trachea, mediastinum, and other respiratory; pleura	951	6	664	69.8	25	2	71	10.0	29.4	25.7	19.2	15.7
Lymphocytic leukemia	276	1.7	174	63	41	9	88	5.8	24.6	21.4	28.3	19.9
Lymphoma	717	4.5	353	49.2	61	26	109	6.1	27.5	29.4	23.3	13.7
Melanoma of the skin; other non‐epithelial skin	1056	6.6	519	49.1	60	25	114	7.7	26.1	24.8	25.4	16.0
Myeloid and monocytic leukemia; Other acute leukemia, aleukemic, subleukemic, NOS	63	0.4	41	65.1	30	10	70	—	22.2	25.4	25.4	—
Myeloma	136	0.9	90	66.2	36	12	72	—	25.0	30.9	24.3	14.0
Oral cavity and pharynx; nose, nasal cavity and middle ear; larynx	518	3.3	262	50.6	58	20	118	8.1	25.9	23.9	22.8	19.3
Ovary	150	0.9	63	42	81	17	194	—	27.3	23.3	21.3	‐
Pancreas	59	0.4	48	81.4	8	0	47	—	28.8	—	—	28.8
Penis; other male genital organs	32	0.2	16	50	52	12	132	—	—	—	—	—
Prostate	5242	32.9	2168	41.4	74	35	125	7.9	26.7	27.4	20.6	17.5
Stomach; retroperitoneum; peritoneum, omentum, and mesentery	226	1.4	161	71.2	15	0	71	16.8	27.9	22.1	19.9	13.3
Thyroid; other endocrine including thymus	207	1.3	91	44	73	30	164	6.8	30.0	22.7	21.7	18.8
Urinary bladder; ureter; other urinary organs	1081	6.8	544	50.3	59	25	123	7.0	25.4	29.3	21.2	17.0
Miscellaneous	331	2.1	248	74.9	24	2	60	16.3	110.7	117.8	82.8	72.4
Total	15935	100	7505	47.1	65	25	124					

Note

29 total types of previous cancer were accounted for in analysis; this table lists 26; due to small cell sizes, Kaposi sarcoma, Mesothelioma, and testis were included within the miscellaneous cancer category. — indicates that at least one cell size per row was ≤11.

### 3.2 Cumulative incidence of death over time and 5‐year risk of death

**FIGURE 1:**
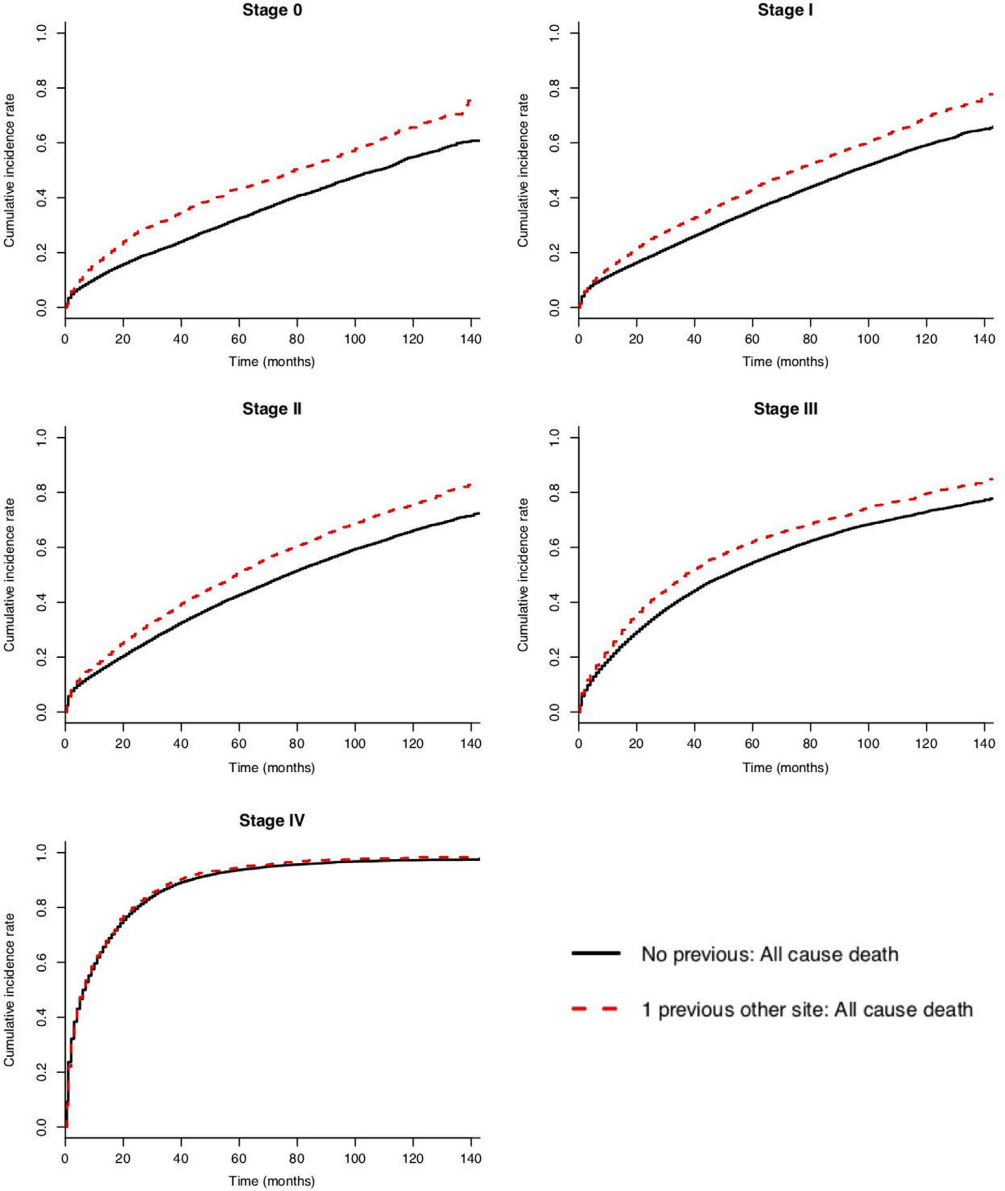
Unadjusted cumulative incidence of all‐cause death and b) cause‐specific death for colorectal cancer (CRC) patients with (n = 15,935) and without (n = 96,834) previous cancer, by CRC stage at diagnosis

**FIGURE 2:**
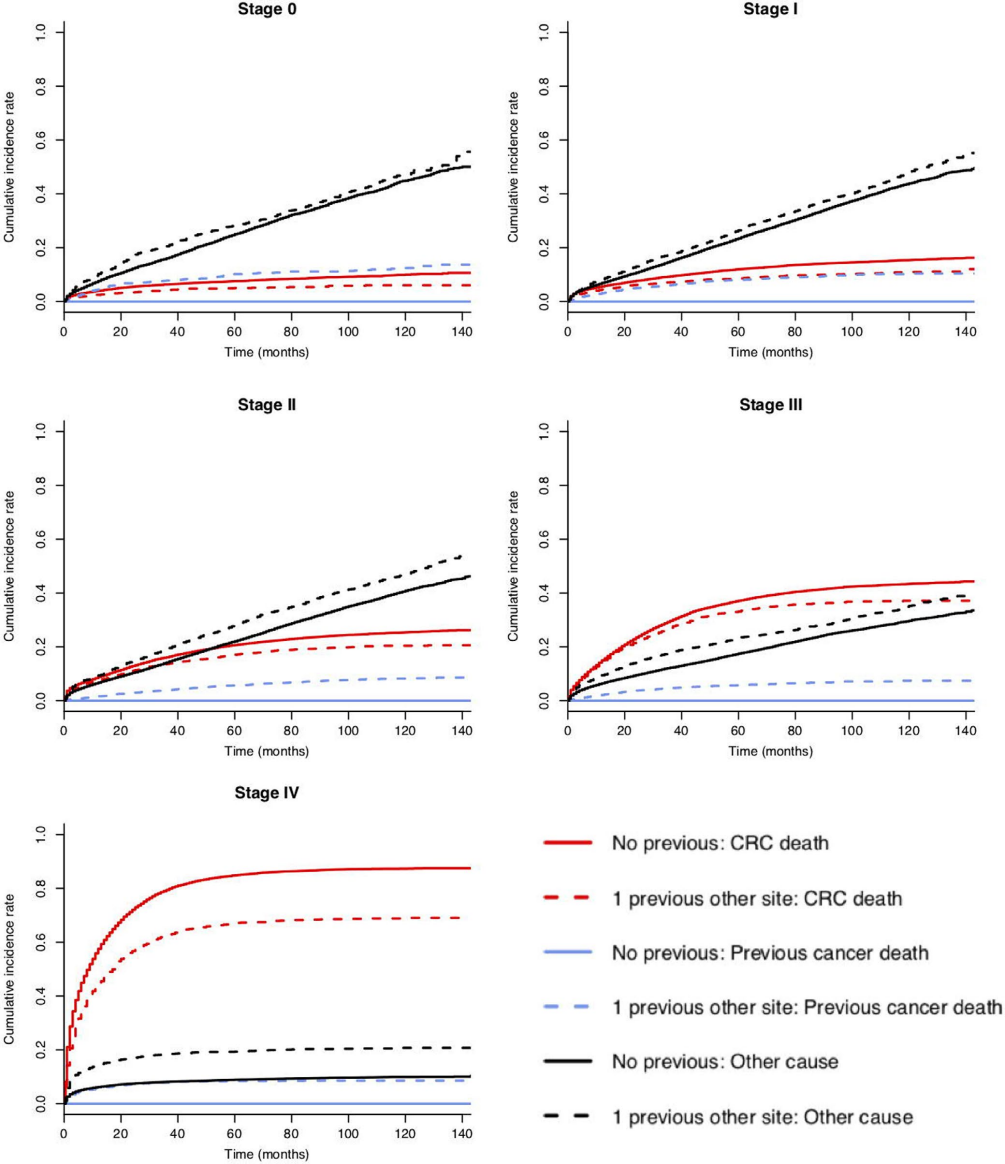
Unadjusted cumulative incidence of cause‐specific death for colorectal cancer (CRC) patients with (n = 15,935) and without (n = 96,834) previous cancer, by CRC stage at diagnosis

Figures 1 and 2 illustrate unadjusted cumulative incidence of all‐cause death cause‐specific death, respectively. For patients with previous cancer compared to those without, the 5‐year risk of death from all causes was as follows: 0.44 versus 0.33 (stage 0), 0.43 versus 0.36 (stage I), 0.51 versus 0.43 (stage II), 0.62 versus 0.55 (stage III), and 0.95 versus 0.94 (stage IV) (see Appendix 4).

### 3.3 Covariate‐adjusted overall and cause‐specific survival

**FIGURE 3:**
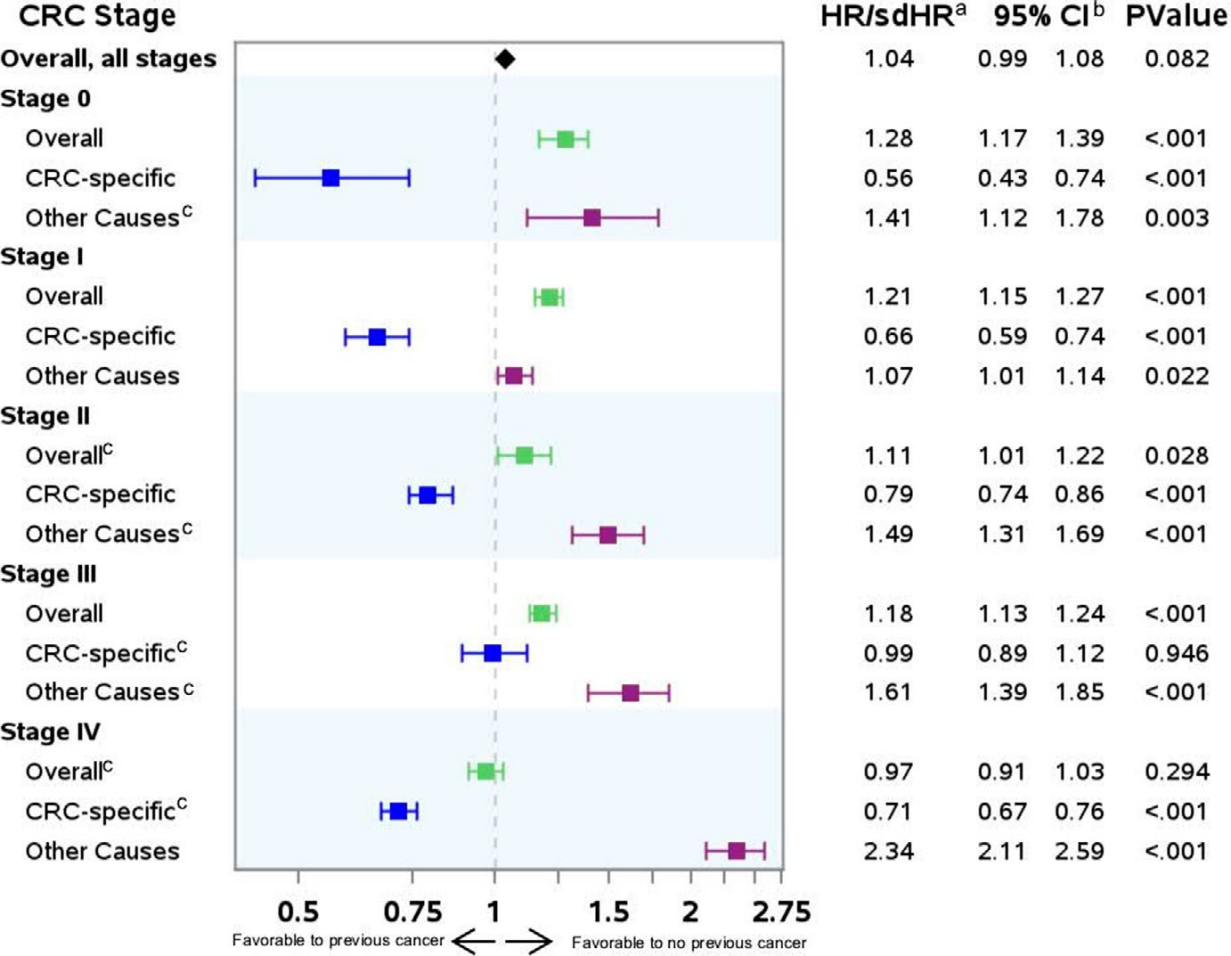
Adjusted association of previous cancer and overall and cause‐specific survival among patients with colorectal cancer (CRC), by CRC stage at diagnosis. *Note for figure*: **^a^** HR = hazard ratios or sdHR = subdistribution hazard ratios generated from Cox proportional hazard models (overall survival) or Fine and Gray proportional subdistribution hazard models (cause‐specific survival). Estimates generated from models adjusted for age, sex, race/ethnicity, year of diagnosis, marital status, U.S. region, neighborhood poverty, urban/rural residence, number of comorbidities and frailties; receipt of surgery, radiation, and chemotherapy for the CRC, and CRC sub‐stage (0, I, IIA, IIB, IIIA, IIIB, IIIC, III not otherwise specified), grade, tumor location, and histology; ^b^CI = confidence interval; **^c^** The previous cancer hazard was not proportional over time in stage II and IV overall regressions and stage 0, II, III, and IV in competing risk regressions. Thus, these models include an interaction term with (log)time; accordingly, for these stages, the previous cancer effect estimate (HR or sdHR) reflects the hazard at time 1 (i.e., 1 month after CRC diagnosis)

Figure 3 illustrates the adjusted association of previous cancer and overall survival and cause‐specific survival. In all CRC stages except stage IV, patients with previous cancer had worse overall survival. For patients with stage IV CRC, there was no difference in overall survival between those with versus without previous cancer.

In competing risk regressions, for patients with all CRC stages except stage III, those with previous cancer had *improved* CRC‐specific survival compared to those without; in stage III, those with versus without previous cancer had equivalent CRC‐specific survival. We observed similar results in unadjusted models (see Appendix 5).

### 3.4 Effect of previous cancer type, timing, and stage on overall survival

**FIGURE 4:**
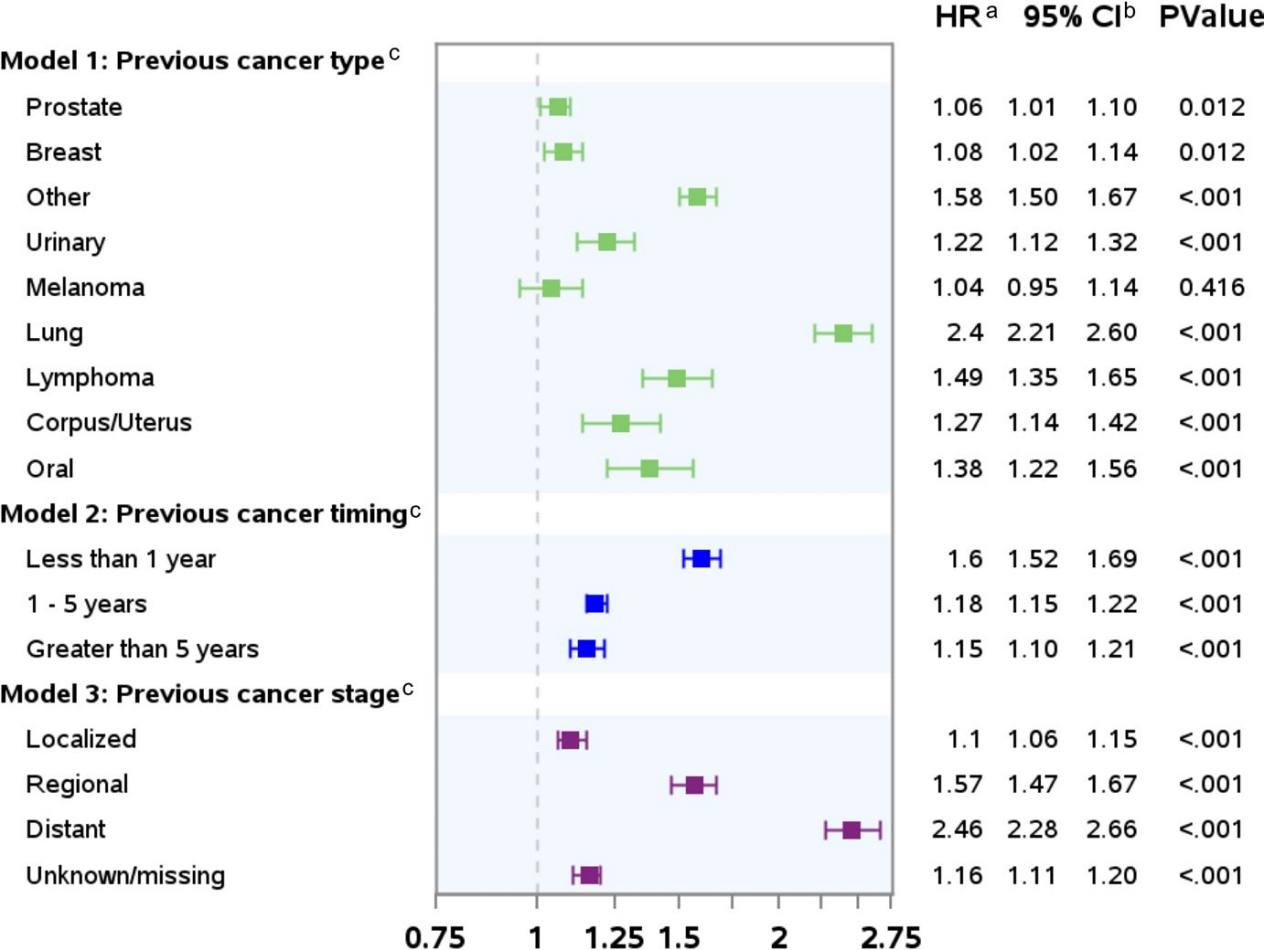
Adjusted association of previous cancer type, timing, and stage and overall survival among patients diagnosed with stage 0–III colorectal cancer (CRC). *Note for figure*: **^a^** HR = hazard ratios generated from Cox proportional hazard models. Estimates generated from models adjusted for age, sex, race/ethnicity, year of diagnosis, marital status, U.S. region, neighborhood poverty, urban/rural residence, number of comorbidities and frailties; receipt of surgery, radiation, and chemotherapy for the colorectal cancer; and CRC sub‐stage (0, I, IIA, IIB, IIIA, IIIB, IIIC, III not otherwise specified), grade, tumor location, and histology. ^b^CI = confidence interval. ^c^The previous cancer hazard was not proportional over time for cancer type, timing, or stage. Thus, these models include an interaction term with (log)time; accordingly, for these stages, the previous cancer effect estimate (HR) reflects the hazard at time 1 (i.e., 1 month after CRC diagnosis)

Because previous cancer was negatively associated with overall survival among patients with stage 0–III CRC, we examined the role of previous cancer type, timing, and stage in this group (n = 91,999). We combined stages 0–III for this analysis and included CRC stage as a covariate. Results from adjusted models are shown in Figure 4 and illustrate worse overall survival for those with all types of previous cancer, except melanoma (adjusted hazard ratio [HR]: 1.04; 0.94–1.14). This association varied in magnitude by type of previous cancer. Relatively small effects were observed among those with previous breast cancer (aHR: 1.07; 95% CI: 1.01–1.14) or prostate cancer (aHR: 1.06, 95% CI: 1.01–1.11). In contrast, patients with previous lung cancer had substantially worse survival (aHR: 2.4; 95% CI: 2.21–2.59). When analyzing time elapsed between diagnosis of previous cancer and CRC, we observed significantly worse survival regardless of timing; the negative association between timing of previous cancer and overall survival was largest for those with previous cancer within a year of their CRC diagnosis. Those with previous cancers diagnosed at a later stage also had worse survival. For example, patients with previous cancers diagnosed at a distant stage had worse survival (aHR: 2.27; 95% CI: 2.29–2.66) compared to those with previous cancers diagnosed at a local stage (aHR: 1.10; 95% CI: 1.06–1.14).

### 3.5 Clinical trial‐type population

Finally, in the subset of 22,943 clinical trial‐type patients (treated patients aged <75 years with no comorbidity or frailty), 16.4% had previous cancer [data not shown]. Among these younger, healthier patients, the direction, magnitude, and significance of the overall survival association were similar to those from the total population. Results from this subset analysis are more generalizable to the types of patients enrolled in clinical trials.

## 4 DISCUSSION

We demonstrated that 14% of patients newly diagnosed with CRC have survived a previous cancer, most often prostate or breast cancer. Compared to those without, patients with previous cancer generally had worse overall survival. This held true for patients diagnosed with stages 0–III CRC, but not for patients diagnosed with stage IV, among whom we observed no difference. Deaths due to previous cancer and other causes, not the incident CRC, drive the overall survival disadvantage for patients with previous cancer. Indeed, we found that CRC‐specific survival, an endpoint of many clinical trials, is *improved* in patients with previous cancer, and at all CRC stages except stage III. The reasons for a CRC‐specific survival advantage are uncertain. Several explanations are plausible, including greater tumor responsiveness, a healthy survivor effect, or other biological differences. Furthermore, it is likely that cancer survivors receive closer medical monitoring and more frequent imaging that may lead to earlier CRC diagnosis and timely treatment.

Prior studies have provided mixed results about the overall impact of previous cancer on survival, perhaps due to differences in cohort selection and analysis methods. In an early report on this topic, patients with incident, resected CRC with previous extracolonic cancer had better 5‐year overall survival than those without previous cancer.^17^ This study included 1,198 patients from a single center in England between 1971 and 1990. In a population‐based study from Finland, women diagnosed with CRC between 1960 and 1993 with previous breast cancer had a slight survival advantage.^16^ However, among 697 patients with gastrointestinal cancer (74% with CRC) and treated with chemotherapy in a London hospital in 2006, previous cancer was *not* associated with differences in overall or disease‐specific survival.^18^ In contrast, a SEER study of CRC patients diagnosed in 2004–2008 found that those with previous cancer had worse overall survival (HR 1.17, 95% CI: 1.15–1.19).^15^ Similar to our findings, the prior SEER study found that, among patients with stage IV CRC, survival was equivalent for those with and without previous cancer.^15^ An additional SEER study of patients with stage IV CRC diagnosed in 1973–2008 found that those with any previous non‐leukemic cancer did not have a significant change in overall survival, however, those with previous leukemia showed worse overall survival.^19^ While our current analysis shares some similarities with these two prior SEER studies, our study focused on older patients and has numerous advantages: It provides stage‐specific estimates of the effect of previous cancer on overall and cause‐specific survival, while accounting for competing risks of death; and importantly, it is the first to examine the role of previous cancer stage and type. Notably, our study also adjusted for many factors important for survival, including frailty, comorbidity, and receipt of chemotherapy; prior SEER studies could not adjust for the impact of these important covariates that are not measured in SEER data but are available in Medicare claims.

### 4.1 Previous cancer characteristics

After observing a negative effect of previous cancer on overall survival among patients with stage 0–III CRC, we examined whether this effect varied by the characteristics of the previous cancer. Results regarding timing and stage were not surprising; patients with previous cancers diagnosed more recently or at a more advanced stage had worse survival, confirming findings from a prior study.^15^ We also illustrated that the impact of previous cancer varied considerably depending on the type of cancer the patient had already survived. Those with previous lung cancer experienced substantially worse survival. In contrast, other types of previous cancer, such as breast or prostate cancer, were associated with a much more modest decrease in survival. Interestingly, we observed that melanoma survivors had equivalent survival when compared to CRC patients with no previous cancer. These findings likely reflect the overall prognosis of patients with these different cancers; while lung cancer survival is very poor, patients with melanoma have good survival; on average, the 5‐year relative survival is 92%.^29^ These findings may also reflect stage distributions of these previous cancers; among those with previous melanoma, 92% were diagnosed with in situ or localized melanoma. To our knowledge, our study is the first to examine the role of previous cancer type and stage on survival, and this evidence should be disseminated to ensure future clinical trial guidelines are evidence‐based.

### 4.2 Implications for trial design

There is increasing interest in modernizing eligibility criteria to improve clinical trial accrual and generalizability of results.^10^, ^11^ Historically, the desire to select favorable and homogeneous subsets of patients is one factor that has driven the tightening of eligibility criteria.^30^, ^31^ We show that CRC patients with previous cancer differs from those without in several respects, and therefore concerns about participant heterogeneity may be warranted. But because, as we show, one in seven older CRC patients has survived a previous cancer, the desire for homogeneity may hamper accrual and representativeness. Inclusion of patients with previous cancer would speed up trial accrual and ensure that trial participants more closely mirror the overall population.

An additional factor driving trial exclusion may be concern about poor outcomes among patients with a previous cancer, such as death prior to the completion of treatment. Our results indicate that this concern is not necessarily warranted. We demonstrated that survival differences varied by CRC stage, type of previous cancer, and by cause of death. Given this complexity, we recommend that trial investigators examine decisions about relaxing exclusion criteria carefully, in light of observed differences in survival, specific endpoints of interest, and the stage of CRC under investigation. Given no difference in overall survival, we recommend that a previous cancer alone should not be an exclusion criterion for patients with stage IV CRC. Likewise, patients with 0–III CRC and a history of melanoma should not be excluded. Lifting these exclusion criteria would result in more rapid accrual and better representation of the rapidly growing population of cancer survivors.

Overall, our results fill several gaps in the literature but additional questions remain. To further inform evidence‐based clinical trial eligibility criteria, more data are needed about other clinically relevant outcomes in this population, including toxicity and tumor response.

### 4.3 Limitations and strengths

Our study faces some limitations. First, there may be misclassification in cause of death. While our competing risk analysis relies on precise classification of causes of death, misclassification would not affect our analysis of overall survival. SEER derives cause of death from death certificates, which can be erroneous. Previous studies indicate that misclassification is more common for patients with previous cancer, particularly those with >2 cancers.^22^ To address this, we limited analysis to patients with only one previous cancer. However, it is still possible that misclassification contributes to CRC‐specific survival advantage as observed in this study. Additional research is needed to fully understand the effects of previous cancer on clinically relevant outcomes and to identify mechanisms leading to improved survival such as more surveillance imaging or earlier treatment among cancer survivors.

Second, it is possible that some CRCs represent misclassified metastases from the earlier tumor. Such misclassification is likely rare, given the strict SEER rules for coding primary cancers^32^ and high rates of microscopic confirmation (over 97% in our sample, regardless of previous cancer status).

Third, we did not include patients ≤65 years of age. The average age of CRC diagnosis is 68 for men and 72 for women,^33^ and the prevalence of previous cancer is more than three times higher among CRC patients ≥65 years compared to those <65 years^3^ ; therefore, our study reflects survival of the majority of CRC patients with previous cancer.

Despite these limitations, our study has several strengths. Our analysis of the competing risks of death is a notable strength of our approach. Accounting for competing causes is important in studies of cancer patients, especially among older patients. This is especially true among cancer patients with previous cancer who face an additional competing risk unique to this population (i.e., death from the previous cancer). Across all stages, approximately one‐fourth of patients died from other causes, with the most common causes of these deaths being heart disease (32.3%), chronic obstructive pulmonary disease (7.3%), and cerebrovascular disease (6.2%). Previous cancer treatment might predispose survivors to death from these other causes, or there may be related causal factors (e.g., smoking, obesity) underlying both the previous cancer diagnosis and these other deaths. We observed, in our stage‐specific analysis, that deaths from other causes were more common than CRC deaths among patients with early stage (0–II) CRC. Failure to account for these competing risks may obscure the true association of previous cancer and survival. For example, it is know that when competing risks are not accounted for, Kaplan–Meier estimates of cause‐specific survival are biased upward and thus risk of death is overestimated.^27^ Prior studies about previous cancer have not accounted for competing risks; thus, the present study overcomes a key limitation in the existing literature.

## 5 CONCLUSIONS

Approximately 14% of CRC patients have previously survived cancer of another type and are at risk of exclusion from clinical trials for that reason alone. Except in stage IV disease, and among patients with stage 0–III CRC and a previous melanoma, those with previous cancer have generally worse overall survival that is not ascribable to their incident CRC. When examined by cause of death, the majority of patients with previous cancer had improved CRC‐specific survival. Given this evidence, patients diagnosed with stage IV CRC with previous cancer and those with previous melanoma should be included in clinical trials.

## CONFLICT OF INTERESTS

DH reports consulting for Braintree labs, Creatics LLC, Abbott Labs, and Macrogenics, Inc., and legal consulting for Noven and Women's Talc Project; AS reports consulting/advisory boards for Bayer, Eisai, Genentech, BMS, Exelixis, Exact Sciences, and GRAIL; and DEG reports research funding from AstraZeneca, BerGenBio, and Karyopharm, Gilead stock ownership and consulting for Samsung Bioepis and Catalyst Pharmaceuticals. SLP, HZ, BM, DX, AT, EAH, and CCM have no disclosures.

## ETHICS APPROVAL AND CONSENT TO PARTICIPATE

The University of Texas Southwestern Medical Center Institutional Review Board approved this study (STU 042018–032).

## AUTHORS’ CONTRIBUTIONS

SLP designed the study and led the writing and CM provided guidance on study design and the analysis plan. HZ, DFH, BM, and DX designed, critically evaluated, and/or performed data analysis. DG, AT, EAH, and AGS provided insight regarding trial design, patient clinical characteristics, and clinical implications of findings. All authors interpreted results of the analysis and provided critical feedback and edits to the manuscript and approved the final manuscript.

## Supporting information

Supplementary Material

## Data Availability

This study used SEER‐MEDICARE data. The Centers for Medicare and Medicaid Services do not allow the redistribution of their data by researchers. SEER‐MEDICARE data are distinct from the publicly available SEER database, and can be obtained by researchers, by following the process described on https://healthcaredelivery.cancer.gov/seermedicare/obtain/requests.html (access requirements include Institutional Review Board approval and the completion of a Data Use Agreement).
