# Supplementary material for: Survival of patients newly diagnosed with colorectal cancer and with a history of previous cancer
Source: Cancer Med. 2021 Jun 30;10(14):4752–67. doi: 10.1002/cam4.4036 (PMC8290226; doi:10.1002/cam4.4036)
Supplement: Supplementary file 1 — Supplementary Material [file CAM4-10-4752-s001.docx]

Contents of Online-Only Appendices

[Appendix 1. Comorbid conditions and frailty-defining diagnoses 2](#_Toc63426716)

[Appendix 2. Diagram of patient selection from linked Surveillance, Epidemiology, and End Results (SEER)-Medicare data of patients diagnosed with colorectal cancer, 2005–2015. 3](#_Toc63426717)

[Appendix 3. Characteristics of patients diagnosed with colorectal cancer (CRC) between 2005-2015 by CRC stage at diagnosis and previous cancer history at time of colorectal cancer diagnosis (n=112,769) 4](#_Toc63426718)

[Appendix 4. Five-year risk of death by cause of death (colorectal cancer [CRC], previous cancer, other causes) and for all causes of death, for patients with and without previous cancer, by CRC stage at diagnosis. 7](#_Toc63426719)

[Appendix 5. Unadjusted association of previous cancer and survival, separately by colorectal cancer (CRC) stage at diagnosis. Survival assessed by Cox proportional hazard models for overall survival and Fine and Gray proportional subdistribution hazard models for CRC-specific death or death from other causes, accounting for the competing risk of death from previous cancer. 8](#_Toc63426720)

# Appendix 1. Comorbid conditions and frailty-defining diagnoses

#

| Comorbid conditions |
| --- |
| Acute Myocardial Infarction |
| History of Myocardial Infarction |
| Congestive Heart Failure |
| Peripheral Vascular Disease |
| Cerebrovascular Disease |
| Chronic Obstructive Pulmonary Disease |
| Dementia |
| Hemiplegia or Paraplegia |
| Diabetes |
| Diabetes with Complications |
| Moderate-Severe Renal Disease |
| Mild Liver Disease |
| Moderate-Severe Liver Disease |
| Peptic Ulcer Disease |
| Rheumatologic Disease |
| Acquired Immunodeficiency Virus (AIDS) |
| Frailty-defining diagnoses |
| Malnutrition: Nutritional marasmus; Other severe protein-calorie malnutrition |
| Dementia: Senile dementia with delusional or depressive features; Senile dementia with delirium |
| Severe vision impairment: Profound impairment, both eyes; Moderate or severe impairment, better eye/lesser eye: profound |
| Decubitus ulcer |
| Incontinence of urine: Incontinence without sensory awareness; Continuous leakage |
| Loss of weight: Abnormal loss of weight and underweight; Feeding difficulties and mismanagement |
| Fecal incontinence: Incontinence of feces |
| Social support needs: Lack of housing; Inadequate housing; Inadequate material resources |
| Difficulty in walking: Difficulty in walking; Abnormality of gait |
| Fall: Fall on stairs or steps, Fall from wheelchair |

# Appendix 2. Diagram of patient selection from linked Surveillance, Epidemiology, and End Results (SEER)-Medicare data of patients diagnosed with colorectal cancer, 2005­–2015.


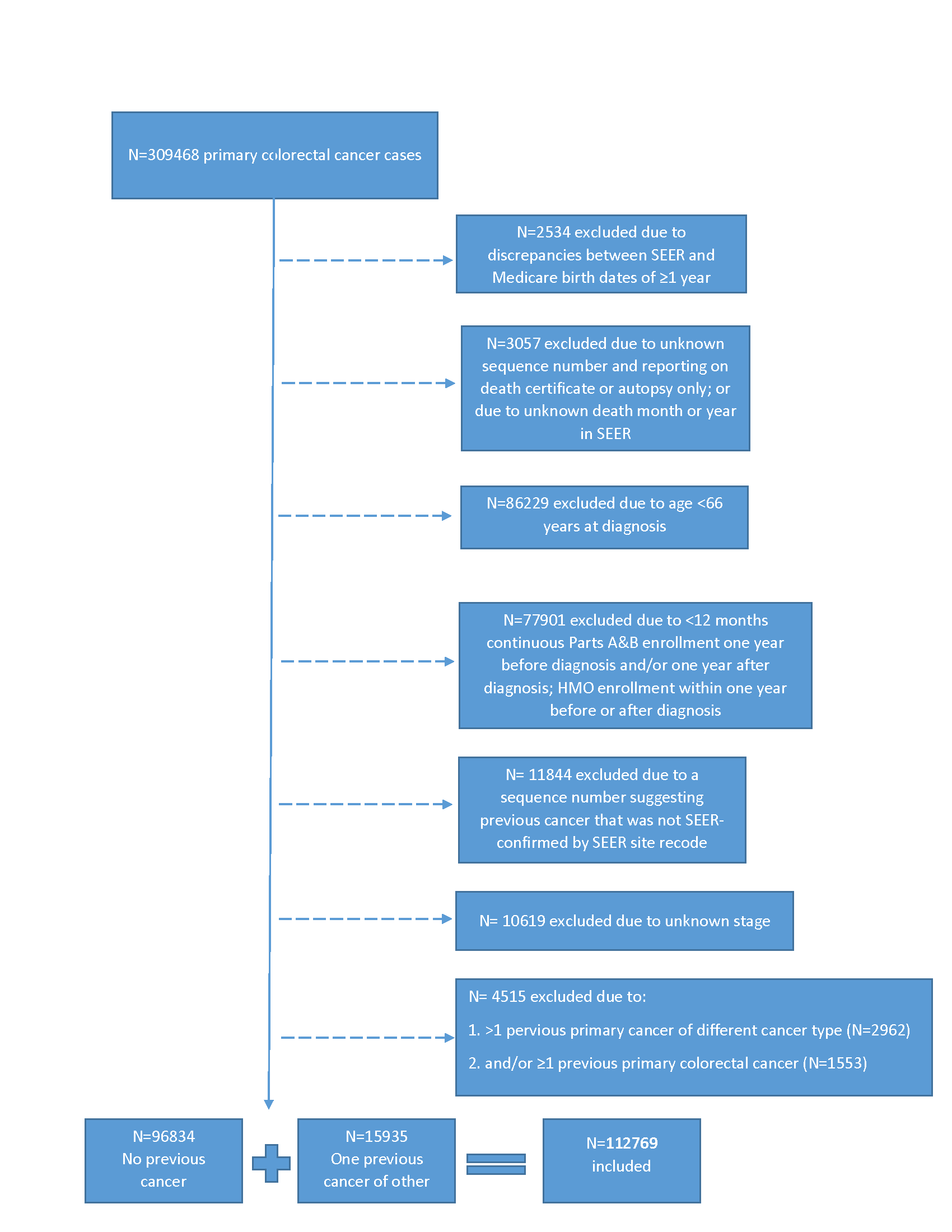


# Appendix 3. Characteristics of patients diagnosed with colorectal cancer (CRC) between 2005-2015 for those with and without previous cancer by CRC stage at diagnosis (n=112,769)

|  | **Stage 0** | | **Stage I** | | **Stage II** | | **Stage III** | | **Stage IV** | |
| --- | --- | --- | --- | --- | --- | --- | --- | --- | --- | --- |
|  | **No previous (n=6733)** | **Previous cancer (n=1246)** | **No previous (n=22509)** | **Previous cancer (n=4204)** | **No previous (n=26513)** | **Previous cancer (n=4292)** | **No previous (n=22957)** | **Previous cancer (n=3545)** | **No previous (n=18122)** | **Previous cancer (n=2648)** |
| **Sex** |  |  |  |  |  |  |  |  |  |  |
| Male | 3371 (50.1%) | 762 (61.2%) | 10566 (46.9%) | 2495 (59.3%) | 11914 (44.9%) | 2440 (56.8%) | 10553 (46.0%) | 1897 (53.5%) | 8579 (47.3%) | 1557 (58.8%) |
| Female | 3362 (49.9%) | 484 (38.8%) | 11943 (53.1%) | 1709 (40.7%) | 14599 (55.1%) | 1852 (43.2%) | 12404 (54.0%) | 1648 (46.5%) | 9543 (52.7%) | 1091 (41.2%) |
| **Age** |  |  |  |  |  |  |  |  |  |  |
| 66-70 | 1410 (20.9%) | 214 (17.2%) | 3936 (17.5%) | 529 (12.6%) | 3804 (14.3%) | 439 (10.2%) | 3892 (17.0%) | 436 (12.3%) | 3147 (17.4%) | 337 (12.7%) |
| 70-75 | 1707 (25.4%) | 338 (27.1%) | 4953 (22.0%) | 929 (22.1%) | 5364 (20.2%) | 756 (17.6%) | 5113 (22.3%) | 751 (21.2%) | 3953 (21.8%) | 524 (19.8%) |
| 75-80 | 1498 (22.2%) | 266 (21.3%) | 5052 (22.4%) | 1053 (25.0%) | 5785 (21.8%) | 938 (21.9%) | 5026 (21.9%) | 793 (22.4%) | 3754 (20.7%) | 582 (22.0%) |
| 80-85 | 1168 (17.3%) | 268 (21.5%) | 4540 (20.2%) | 920 (21.9%) | 5598 (21.1%) | 1095 (25.5%) | 4530 (19.7%) | 833 (23.5%) | 3475 (19.2%) | 573 (21.6%) |
| >85 | 950 (14.1%) | 160 (12.8%) | 4028 (17.9%) | 773 (18.4%) | 5962 (22.5%) | 1064 (24.8%) | 4396 (19.1%) | 732 (20.6%) | 3793 (20.9%) | 632 (23.9%) |
| **Race/ethnicity** |  |  |  |  |  |  |  |  |  |  |
| Non-Hispanic  White | 5130 (76.2%) | 962 (77.2%) | 18076 (80.3%) | 3515 (83.6%) | 21456 (80.9%) | 3630 (84.6%) | 17966 (78.3%) | 2926 (82.5%) | 13829 (76.3%) | 2107 (79.6%) |
| Hispanic white | 294 (4.4%) | - | 830 (3.7%) | - | 1024 (3.9%) | - | 953 (4.2%) | - | 779 (4.3%) | - |
| Black | 770 (11.4%) | 154 (12.4%) | 1879 (8.3%) | 344 (8.2%) | 2059 (7.8%) | 295 (6.9%) | 2067 (9.0%) | 296 (8.3%) | 2097 (11.6%) | 300 (11.3%) |
| Other | 523 (7.8%) | 92  (7.4%) | 1680 (7.5%) | 222 (5.3%) | 1919 (7.2%) | 223 (5.2%) | 1924 (8.4%) | 197 (5.6%) | 1371 (7.6%) | 145 (5.5%) |
| Unknown | 16 (0.2%) | - | 44 (0.2%) | - | 55 (0.2%) | - | 47 (0.2%) | - | 46 (0.3%) | - |
| **Marital status** |  |  |  |  |  |  |  |  |  |  |
| Married/Unmarried  or domestic partner | 3254 (48.3%) | 678 (54.4%) | 11316 (50.3%) | 2289 (54.4%) | 12387 (46.7%) | 2289 (53.3%) | 11073 (48.2%) | 1845 (52.0%) | 8124 (44.8%) | 1314 (49.6%) |
| Separated/  Divorced  /Widowed | 2257 (33.5%) | 379 (30.4%) | 8068 (35.8%) | 1363 (32.4%) | 10551 (39.8%) | 1488 (34.7%) | 8848 (38.5%) | 1296 (36.6%) | 7273 (40.1%) | 1000 (37.8%) |
| Single | 596 (8.9%) | 94  (7.5%) | 1851 (8.2%) | 327 (7.8%) | 2470 (9.3%) | 338 (7.9%) | 2079 (9.1%) | 261 (7.4%) | 1974 (10.9%) | 233 (8.8%) |
| Unknown | 626 (9.3%) | 95  (7.6%) | 1274 (5.7%) | 225 (5.4%) | 1105 (4.2%) | 177 (4.1%) | 957 (4.2%) | 143 (4.0%) | 751 (4.1%) | 101 (3.8%) |
| **Medicaid** |  |  |  |  |  |  |  |  |  |  |
| Yes | 1287 (19.1%) | 187 (15.0%) | 3928 (17.5%) | 520 (12.4%) | 4891 (18.4%) | 522 (12.2%) | 4429 (19.3%) | 485 (13.7%) | 3763 (20.8%) | 406 (15.3%) |
| No | 5446 (80.9%) | 1059 (85.0%) | 18581 (82.5%) | 3684 (87.6%) | 21622 (81.6%) | 3770 (87.8%) | 18528 (80.7%) | 3060 (86.3%) | 14359 (79.2%) | 2242 (84.7%) |
| **Poverty** |  |  |  |  |  |  |  |  |  |  |
| 0% to <5% | 1583 (23.5%) | 373 (29.9%) | 5232 (23.2%) | 1095 (26.0%) | 6016 (22.7%) | 1093 (25.5%) | 5238 (22.8%) | 906 (25.6%) | 3867 (21.3%) | 635 (24.0%) |
| 5% to <10% | 1678 (24.9%) | 300 (24.1%) | 5872 (26.1%) | 1146 (27.3%) | 6919 (26.1%) | 1222 (28.5%) | 5946 (25.9%) | 1001 (28.2%) | 4546 (25.1%) | 704 (26.6%) |
| 10% to <20% | 1948 (28.9%) | 312 (25.0%) | 6696 (29.7%) | 1169 (27.8%) | 7940 (29.9%) | 1195 (27.8%) | 6832 (29.8%) | 955 (26.9%) | 5364 (29.6%) | 762 (28.8%) |
| 20% to 100% | 1503 (22.3%) | - | 4659 (20.7%) | 780 (18.6%) | 5579 (21.0%) | - | 4902 (21.4%) | - | 4304 (23.8%) | - |
| Unknown | 21 (0.3%) | - | 50 (0.2%) | 14 (0.3%) | 59 (0.2%) | - | 39 (0.2%) | - | 41 (0.2%) | - |
| **Urban-rural indicator** |  |  |  |  |  |  |  |  |  |  |
| Metro | 5505 (81.8%) | 1036 (83.1%) | 18214 (80.9%) | 3466 (82.4%) | 21709 (81.9%) | 3576 (83.3%) | 18846 (82.1%) | 2957 (83.4%) | 14831 (81.8%) | 2190 (82.7%) |
| Urban | 1072 (15.9%) | 184 (14.8%) | 3751 (16.7%) | 657 (15.6%) | 4198 (15.8%) | 620 (14.4%) | 3542 (15.4%) | 511 (14.4%) | 2877 (15.9%) | 404 (15.3%) |
| Rural or Unknown | 156 (2.3%) | 26  (2.1%) | 544 (2.4%) | 81  (1.9%) | 602 (2.3%) | 96  (2.2%) | 569 (2.5%) | 77  (2.2%) | 414 (2.3%) | 54  (2.0%) |
| **Region** |  |  |  |  |  |  |  |  |  |  |
| Northeast | 1849 (27.5%) | 370 (29.7%) | 4721 (21.0%) | 868 (20.6%) | 5874 (22.2%) | 876 (20.4%) | 4865 (21.2%) | 709 (20.0%) | 3928 (21.7%) | 543 (20.5%) |
| South | 1935 (28.7%) | 312 (25.0%) | 6071 (27.0%) | 892 (21.2%) | 6616 (25.0%) | 814 (19.0%) | 5992 (26.1%) | 758 (21.4%) | 4611 (25.4%) | 521 (19.7%) |
| Midwest | 516 (7.7%) | 117 (9.4%) | 2997 (13.3%) | 747 (17.8%) | 3645 (13.7%) | 862 (20.1%) | 3030 (13.2%) | 635 (17.9%) | 2433 (13.4%) | 490 (18.5%) |
| West | 2433 (36.1%) | 447 (35.9%) | 8720 (38.7%) | 1697 (40.4%) | 10378 (39.1%) | 1740 (40.5%) | 9070 (39.5%) | 1443 (40.7%) | 7150 (39.5%) | 1094 (41.3%) |
| **Tumor location** |  |  |  |  |  |  |  |  |  |  |
| Proximal colon (cecum, ascending) | 2099 (31.2%) | 396 (31.8%) | 8063 (35.8%) | 1513 (36.0%) | 10846 (40.9%) | 1804 (42.0%) | 9270 (40.4%) | 1571 (44.3%) | 5977 (33.0%) | 924 (34.9%) |
| Transverse colon (hepatic flexure, transverse colon, splenic flexure) | 708 (10.5%) | - | 2819 (12.5%) | 591 (14.1%) | 4519 (17.0%) | 810 (18.9%) | 3277 (14.3%) | - | 2256 (12.4%) | 374 (14.1%) |
| Distal colon (descending colon, sigmoid colon, overlapping lesion, Colon NOS) | 2028 (30.1%) | 359 (28.8%) | 5402 (24.0%) | 970 (23.1%) | 5980 (22.6%) | 908 (21.2%) | 5135 (22.4%) | 736 (20.8%) | 5687 (31.4%) | 754 (28.5%) |
| Rectum (rectosigmoid junction, rectum) | 1854 (27.5%) | 324 (26.0%) | 6109 (27.1%) | 1097 (26.1%) | 4884 (18.4%) | 707 (16.5%) | 5157 (22.5%) | 703 (19.8%) | 3892 (21.5%) | 549 (20.7%) |
| Unknown | 44  (0.7%) | - | 116 (0.5%) | 33  (0.8%) | 284 (1.1%) | 63  (1.5%) | 118 (0.5%) | 15  (0.4%) | 310 (1.7%) | 47  (1.8%) |
| **Grade** |  |  |  |  |  |  |  |  |  |  |
| Well differentiated | 739 (11.0%) | 155 (12.4%) | 3279 (14.6%) | 600 (14.3%) | 1854 (7.0%) | 279 (6.5%) | 1174 (5.1%) | 160 (4.5%) | 710 (3.9%) | 110 (4.2%) |
| Moderately  differentiated | 1038 (15.4%) | 197 (15.8%) | 14868 (66.1%) | 2764 (65.7%) | 18708 (70.6%) | 3051 (71.1%) | 14300 (62.3%) | 2157 (60.8%) | 7707 (42.5%) | 1027 (38.8%) |
| Poorly differentiated | 211 (3.1%) | 30  (2.4%) | 1718 (7.6%) | 318 (7.6%) | 4272 (16.1%) | 703 (16.4%) | 5724 (24.9%) | 944 (26.6%) | 3585 (19.8%) | 584 (22.1%) |
| Undifferentiated | 139 (2.1%) | 18  (1.4%) | 203 (0.9%) | 44  (1.0%) | 592 (2.2%) | 111 (2.6%) | 893 (3.9%) | 164 (4.6%) | 536 (3.0%) | 81  (3.1%) |
| Not determined | 4606 (68.4%) | 846 (67.9%) | 2441 (10.8%) | 478 (11.4%) | 1087 (4.1%) | 148 (3.4%) | 866 (3.8%) | 120 (3.4%) | 5584 (30.8%) | 846 (31.9%) |
| **Histology** |  |  |  |  |  |  |  |  |  |  |
| Mucinous  adenocarcinoma | 6270 (93.1%) | 1174 (94.2%) | 20873 (92.7%) | 3912 (93.1%) | 22962 (86.6%) | 3676 (85.6%) | 19810 (86.3%) | 3007 (84.8%) | 13921 (76.8%) | 2002 (75.6%) |
| Other adenocarcinoma | - | - | 1136 (5.0%) | 212 (5.0%) | 3078 (11.6%) | 547 (12.7%) | 2655 (11.6%) | 442 (12.5%) | 1847 (10.2%) | 300 (11.3%) |
| Non-adenocarcinoma | 424 (6.3%) | 64  (5.1%) | 408 (1.8%) | 63  (1.5%) | 427 (1.6%) | - | 433 (1.9%) | - | 1331 (7.3%) | 240 (9.1%) |
| Unknown | - | - | 92  (0.4%) | 17  (0.4%) | 46  (0.2%) | - | 59  (0.3%) | - | 1023 (5.6%) | 106 (4.0%) |
| **Surgery** |  |  |  |  |  |  |  |  |  |  |
| No surgery | 737 (10.9%) | 149 (12.0%) | 2038 (9.1%) | 412 (9.8%) | 1141 (4.3%) | 182 (4.2%) | 798 (3.5%) | 136 (3.8%) | 8896 (49.1%) | 1372 (51.8%) |
| Local tumor  excision | 3050 (45.3%) | 580 (46.5%) | 3217 (14.3%) | 672 (16.0%) | 251 (0.9%) | 37  (0.9%) | 100 (0.4%) | 21  (0.6%) | 198 (1.1%) | 37  (1.4%) |
| Partial colectomy  or proctectomy | 1274 (18.9%) | 217 (17.4%) | 7974 (35.4%) | 1393 (33.1%) | 9757 (36.8%) | 1546 (36.0%) | 9126 (39.8%) | 1299 (36.6%) | 3584 (19.8%) | 471 (17.8%) |
| Subtotal colectomy | 1525 (22.6%) | 285 (22.9%) | 8393 (37.3%) | 1546 (36.8%) | 13977 (52.7%) | 2298 (53.5%) | 11460 (49.9%) | 1857 (52.4%) | 4703 (26.0%) | 677 (25.6%) |
| Total colectomy or proctectomy | 54  (0.8%) | - | 607 (2.7%) | 131 (3.1%) | 916 (3.5%) | 191 (4.5%) | 1104 (4.8%) | 176 (5.0%) | 341 (1.9%) | - |
| Proctocolectomy | 21  (0.3%) | 0  (0.0%) | 110 (0.5%) | 21  (0.5%) | 264 (1.0%) | - | 228 (1.0%) | 30  (0.8%) | 162 (0.9%) | - |
| Other/not specified | 72  (1.1%) | - | 170 (0.8%) | 29  (0.7%) | 207 (0.8%) | - | 141 (0.6%) | 26  (0.7%) | 238 (1.3%) | 24  (0.9%) |
| **Chemotherapy** |  |  |  |  |  |  |  |  |  |  |
| Yes | 311 (4.6%) | 224 (18.0%) | 1760 (7.8%) | 730 (17.4%) | 4836 (18.2%) | 1035 (24.1%) | 11645 (50.7%) | 1794 (50.6%) | 8319 (45.9%) | 1291 (48.8%) |
| No | 6422 (95.4%) | 1022 (82.0%) | 20749 (92.2%) | 3474 (82.6%) | 21677 (81.8%) | 3257 (75.9%) | 11312 (49.3%) | 1751 (49.4%) | 9803 (54.1%) | 1357 (51.2%) |
| **Radiation** |  |  |  |  |  |  |  |  |  |  |
| Yes | 209 (3.1%) | 143 (11.5%) | 1807 (8.0%) | 507 (12.1%) | 3261 (12.3%) | 570 (13.3%) | 3525 (15.4%) | 537 (15.1%) | 1914 (10.6%) | 309 (11.7%) |
| No | 6524 (96.9%) | 1103 (88.5%) | 20702 (92.0%) | 3697 (87.9%) | 23252 (87.7%) | 3722 (86.7%) | 19432 (84.6%) | 3008 (84.9%) | 16208 (89.4%) | 2339 (88.3%) |
| **Comorbidity count** |  |  |  |  |  |  |  |  |  |  |
| 0 | 2876 (42.7%) | 502 (40.3%) | 9823 (43.6%) | 1736 (41.3%) | 12095 (45.6%) | 1732 (40.4%) | 10866 (47.3%) | 1531 (43.2%) | 8804 (48.6%) | 1151 (43.5%) |
| 1 | 1942 (28.8%) | 367 (29.5%) | 6425 (28.5%) | 1218 (29.0%) | 7619 (28.7%) | 1339 (31.2%) | 6558 (28.6%) | 1043 (29.4%) | 4928 (27.2%) | 755 (28.5%) |
| 2 | 936 (13.9%) | 198 (15.9%) | 3160 (14.0%) | 648 (15.4%) | 3675 (13.9%) | 648 (15.1%) | 3030 (13.2%) | 525 (14.8%) | 2350 (13.0%) | 371 (14.0%) |
| ≥ 3 | 979 (14.5%) | 179 (14.4%) | 3101 (13.8%) | 602 (14.3%) | 3124 (11.8%) | 573 (13.4%) | 2503 (10.9%) | 446 (12.6%) | 2040 (11.3%) | 371 (14.0%) |
| **Frailty count** |  |  |  |  |  |  |  |  |  |  |
| 0 | 5201 (77.2%) | 955 (76.6%) | 17448 (77.5%) | 3250 (77.3%) | 19412 (73.2%) | 3174 (74.0%) | 17080 (74.4%) | 2603 (73.4%) | 12242 (67.6%) | 1804 (68.1%) |
| 1 | 1203 (17.9%) | 243 (19.5%) | 3970 (17.6%) | 762 (18.1%) | 5642 (21.3%) | 916 (21.3%) | 4727 (20.6%) | 728 (20.5%) | 4584 (25.3%) | 674 (25.5%) |
| 2 | 249 (3.7%) | 34  (2.7%) | 866 (3.8%) | 150 (3.6%) | 1171 (4.4%) | 161 (3.8%) | 930 (4.1%) | 170 (4.8%) | 1067 (5.9%) | 139 (5.2%) |
| ≥ 3 | 80  (1.2%) | 14  (1.1%) | 225 (1.0%) | 42  (1.0%) | 288 (1.1%) | 41  (1.0%) | 220 (1.0%) | 44  (1.2%) | 229 (1.3%) | 31  (1.2%) |
| **Cause of death** |  |  |  |  |  |  |  |  |  |  |
| Alive | 3806 (56.5%) | 590 (47.4%) | 12404 (55.1%) | 2060 (49.0%) | 13022 (49.1%) | 1834 (42.7%) | 9282 (40.4%) | 1267 (35.7%) | 1405 (7.8%) | 191 (7.2%) |
| Died of colorectal cancer | 586 (8.7%) | 66  (5.3%) | 2944 (13.1%) | 386 (9.2%) | 5744 (21.7%) | 743 (17.3%) | 8650 (37.7%) | 1149 (32.4%) | 15087 (83.3%) | 1728 (65.3%) |
| Died of previous cancer | 0  (0%) | 135 (10.8%) | 0  (0%) | 355 (8.4%) | 0  (0%) | 268 (6.2%) | 0  (0%) | 211 (6.0%) | 0  (0%) | 216 (8.2%) |
| Died of other causes | 2341 (34.8%) | 455 (36.5%) | 7161 (31.8%) | 1403 (33.4%) | 7747 (29.2%) | 1447 (33.7%) | 5025 (21.9%) | 918 (25.9%) | 1630 (9.0%) | 513 (19.4%) |

Note: – indicates that at least one cell size per column with a known covariate was ≤11.

# Appendix 4. Five-year risk of death by cause of death (colorectal cancer [CRC], previous cancer, other causes) and for all causes of death, for patients with and without previous cancer, by CRC stage at diagnosis.

|  | **No previous cancer** | | | **Previous cancer** | | |
| --- | --- | --- | --- | --- | --- | --- |
|  | Cumulative  Incidence | 95% Confidence Interval | | Cumulative  Incidence | 95% Confidence Interval | |
| **Stage 0** | | | | | | |
| CRC Death | 0.08 | 0.07 | 0.08 | 0.05 | 0.04 | 0.06 |
| Previous Cancer Death | . | . | . | 0.10 | 0.09 | 0.12 |
| Other Causes | 0.25 | 0.24 | 0.26 | 0.28 | 0.26 | 0.31 |
| All Causes | 0.33 | 0.32 | 0.34 | 0.44 | 0.41 | 0.46 |
| **Stage 1** | | | | | | |
| CRC Death | 0.12 | 0.12 | 0.13 | 0.09 | 0.08 | 0.10 |
| Previous Cancer Death | . | . | . | 0.08 | 0.07 | 0.09 |
| Other Causes | 0.24 | 0.23 | 0.24 | 0.27 | 0.25 | 0.28 |
| All Causes | 0.36 | 0.35 | 0.36 | 0.43 | 0.41 | 0.45 |
| **Stage II** | | | | | | |
| CRC Death | 0.21 | 0.20 | 0.21 | 0.17 | 0.16 | 0.18 |
| Previous Cancer Death | . | . | . | 0.06 | 0.05 | 0.06 |
| Other Causes | 0.22 | 0.22 | 0.23 | 0.28 | 0.27 | 0.29 |
| All Causes | 0.43 | 0.42 | 0.43 | 0.51 | 0.49 | 0.52 |
| **Stage III** | | | | | | |
| CRC Death | 0.37 | 0.37 | 0.38 | 0.33 | 0.32 | 0.35 |
| Previous Cancer Death | . | . | . | 0.06 | 0.05 | 0.07 |
| Other Causes | 0.18 | 0.17 | 0.18 | 0.23 | 0.22 | 0.25 |
| All Causes | 0.55 | 0.54 | 0.55 | 0.62 | 0.60 | 0.64 |
| **Stage IV** | | | | | | |
| CRC Death | 0.85 | 0.84 | 0.85 | 0.67 | 0.65 | 0.69 |
| Previous Cancer Death | . | . | . | 0.08 | 0.07 | 0.10 |
| Other Causes | 0.09 | 0.09 | 0.09 | 0.19 | 0.18 | 0.21 |
| All Causes | 0.94 | 0.93 | 0.94 | 0.95 | 0.94 | 0.96 |

Appendix 5. Unadjusted association of previous cancer and overall and cause-specific survival among patients with colorectal cancer (CRC), by CRC stage at diagnosis.

**
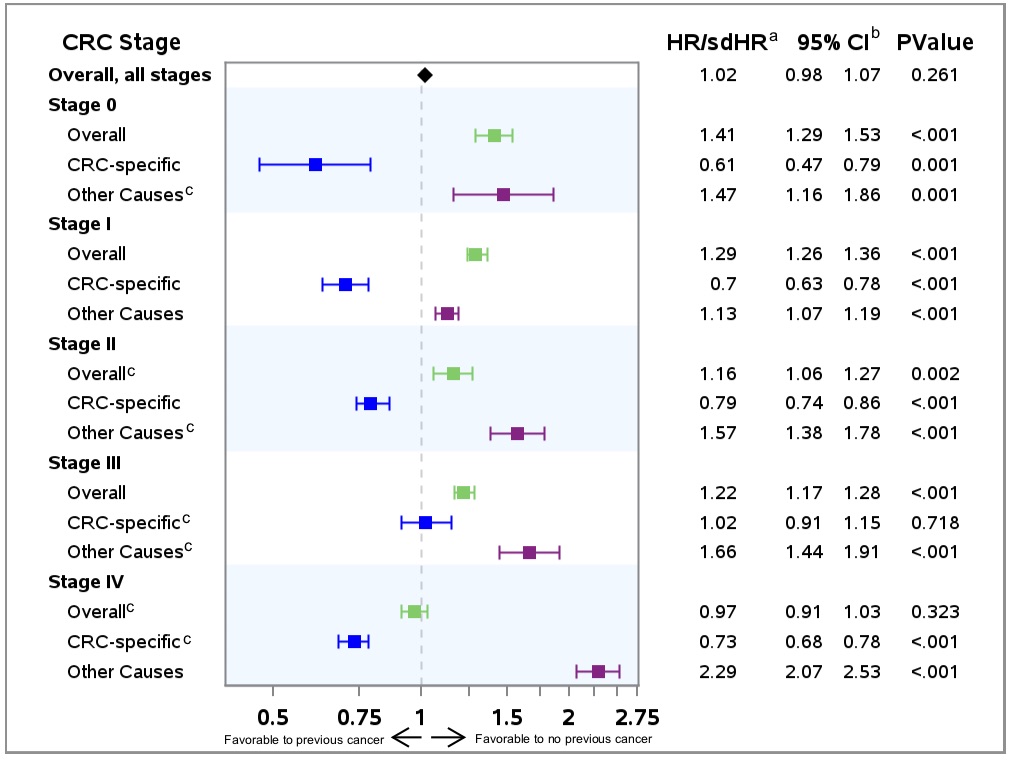
**

**^a^**HR=hazard ratios or sdHR=subdistribution hazard ratios generated from Cox proportional hazard models (overall survival) or Fine and Gray proportional subdistribution hazard models (cause-specific survival); ^b^CI=confidence interval; **^c^**The previous cancer hazard was not proportional over time in stages II and IV overall models and stages 0, II, III, and IV in competing-risk models. Thus, these models include an interaction term with time; accordingly, for these stages, the previous cancer effect estimate (HR or sdHR) demonstrates the hazard at time 1 (i.e., 1 month after CRC diagnosis).
